# Emerging and Re-Emerging Diseases: Novel Challenges in Today’s World or More of the Same?

**DOI:** 10.3390/ani11082382

**Published:** 2021-08-12

**Authors:** João R. Mesquita

**Affiliations:** 1 Instituto de Ciências Biomédicas Abel Salar (ICBAS), Universidade do Porto, 4050-313 Porto, Portugal; jrmesquita@icbas.up.pt; Tel.: +351-220-428-000; 2 Epidemiology Research Unit (EPIUnit), Instituto de Saúde Pública da Universidade do Porto (ISPUP), 4050-313 Porto, Portugal

## Abstract

More than 61% of all human pathogens are zoonotic, representing 75% of all emerging pathogens during the past decade. Albeit significant technological leaps in diagnostics development and disease surveillance, zoonotic emerging infectious diseases are evermore a matter of concern, particularly in modern days where global warming keeps providing ideal climatic conditions to the introduction of exotic infectious agents or disease vectors in new territories. Worryingly, the 2019 novel coronavirus epidemic acts as an extreme reminder of the role animal reservoirs play in public health, accounting for over 4,200,000 deaths worldwide until today. In this Special Issue, we approach a myriad of zoonotic infectious diseases and their complex mechanisms. This Special Issue is composed of three reviews on zoonotic diseases of African Lions, hemogregarine classification, and hepatitis E virus in Brazil, followed by one letter and one opinion piece that broadens the spectrum of disease emergence to mechanistic aspects of emerging non-communicable diseases. The Special Issue is completed by six research papers covering a wide array of emerging and re-emerging diseases of poultry, bovine, poultry and tortoises, of various nature such as parasitic, bacterial, and viral. This is a brief but assertive collection that showcases the need to address health at the animal–human–environment interface, in a One Health perspective.

## 1. Introduction

The notion of crossing the species barrier in infectious diseases derives from the relationship between infectious agents, such as viruses and bacteria, and their host species, which is restricted by genetic adaptations that develop through co-evolution [1]. Spillover of these agents is a reality that occurs frequently, potentially leading to the development of severe disease in the new hosts [1]. Pathogens cross the species barrier frequently that it is today known that over 61% of all human infectious diseases are of zoonotic origin, representing 75% of all emerging pathogens during the past 10 years [2]. Although substantial developments in medical/environmental surveillance and in diagnostic methods have been recently achieved, zoonotic emerging and re-emerging diseases are still a major global concern. In fact, such threats are expanding under global warming conditions, particularly in less developed regions. However, this has not started today. Emerging (and re-emerging) transmissible diseases have been impacting human populations since the Agricultural revolution, when hunter-gatherers settled and started crop cultivation and animal domestication, circa 12,000 years ago, reflecting man’s first steps in nature’s manipulation [3,4]. Since then, a vast number of animal and human diseases have circulated on the earth’s surface [4], reaching to the current 2019 novel coronavirus epidemic as an extreme reminder of the role animal reservoirs play in public health [5], shedding SARS-CoV-2 to humans where it adapted and became transmissible by air [6] and surfaces [7].

This Special Issue of Animals: “Emerging and Re-Emerging Diseases—Novel Challenges in Today’s World”, presents a total of 11 manuscripts focusing on an important group of aspects related to diseases that are found to significantly imbalance ecosystems where humans/animals, pathogens, and the environment interact.

## 2. Reviews on Wildlife, Taxonomy, and Public Health

It first starts with three interesting reviews, the first on zoonotic diseases of African Lions, (*Panthera leo*) that are bred in captivity on commercial farms across South Africa and often have close contact with farm staff, tourists, and other industry workers, hence posing a potential risk of disease interchange between lions and humans [8]. The systematic review describes a total of 63 pathogenic organisms, with several known pathogens that can be transmitted from lions to other species, including humans. The second review [9] is focused on hemogregarines, apicomplexan blood parasites with an obligatory heteroxenous life cycle that are common blood parasites of fish, amphibians, lizards, snakes, turtles, tortoises, crocodilians, birds, and mammals. This work recognizes that proper classification for the hemogregarine complex is available and further develops on evolutionary relationships producing a reflection on the criteria of generic and unique diagnosis of these parasites. The last review proposes a systematic presentation of hepatitis E virus in humans, animals, and environment of Brazil, the fourth largest pig producer in the world [10]. The review shows that hepatitis E virus genotype 3 was the only retrieved genotype in humans, animals, and environment in Brazil. The South region of Brazil showed the highest human seroprevalence and also the highest density of pigs and related industry, suggesting a zoonotic link and allowing to infer that hepatitis E virus epidemiology in Brazil is similar to that of industrialized countries.

## 3. Letters and Opinions

These reviews are followed by one letter and one opinion piece that broadens the spectrum of disease emergence to mechanistic aspects of emerging non-communicable diseases by developing the topic of trefoil factor family member 2 (TFF2), discussing, particularly, the role of high-fat diet-induced TFF2 in counteracting immune-mediated damage [11] and as an inflammatory-induced and anti-inflammatory tissue repair factor [12].

## 4. A Wide Diversity of Original Research

The Special Issue is completed by six research papers covering a wide array of emerging and re-emerging diseases of poultry, bovine, poultry and tortoises, of various nature such as parasitic, bacterial, and viral. The first is a descriptive pathological study of avian schistosomes infection in Whooper Swans (*Cygnus cygnus*) from rescue/rehabilitation centers in Honshu, Japan, reporting that swans most likely died from obstructive phlebitis associated with *Allobilharzia visceralis* [13]. Additionally, more avian pathogens were assessed, initially bacteria, such as *Salmonella* Minnesota, with the genomic characterization of clonal lineages associated with poultry production in Brazil, demonstrating the dissemination of two distinct *S. Minnesota* lineages with high resistance to antibiotics and important virulence genetic clusters in Brazilian poultry farms [14]. A study on a viral pathogen of avian origin, specifically, avian influenza H9N2 in broiler chicken, compared the effectiveness of two different vaccination regimes, ultimately suggesting the use of a vaccine prepared from a recently circulating H9N2 that showed significantly higher protection than the other and was found to be more suitable for birds in the Middle East [15]. This Issue then presents a paper on bovine diseases, initially presenting a molecular approach on the characterization of bovine papillomavirus Type 1 (BPV-1) in cattle from Egypt. In addition, the development of a point-of-need molecular test for BPV-1 diagnosis is described, showing diagnostic utility comparable to PCR-based testing [16]. This article is followed by a study on the first isolation and molecular characterization of bovine respiratory syncytial virus strains in Turkish cattle, a disease with a huge economic burden on livestock industries of countries worldwide [17]. Lastly, the final work reports the molecular detection and characterization of tick-borne agents on *Hyalomma aegyptium* ticks from tortoises of a black market in Doha, Qatar. This study includes the detection of *Hemolivia mauritanica*, *Ehrlichia* spp., and *Candidatus* Midichloria Mitochondrii and highlights the dangers of the international trade of tortoises carrying ticks infected with pathogens of veterinary and medical importance [18].

## 5. Concluding Remarks

This is a brief but assertive collection that showcases the need to address health at the animal–human–environment interface, in a One Health approach. The global perspective highlighted by the content of this Special Issue reinforces the need of joint and wide efforts by stakeholders.

## Data Availability

Not applicable.
